# A Weighted Subdomain Adaptation Network for Partial Transfer Fault Diagnosis of Rotating Machinery

**DOI:** 10.3390/e23040424

**Published:** 2021-04-01

**Authors:** Sixiang Jia, Jinrui Wang, Xiao Zhang, Baokun Han

**Affiliations:** College of Mechanical and Electronic Engineering, Shandong University of Science and Technology, Qingdao 266590, China; sixiang_j@163.com (S.J.); wangjr33@163.com (J.W.); zhangxiao9789@163.com (X.Z.)

**Keywords:** domain adaptation, partial transfer, fault diagnosis, subdomain, rotating machinery

## Abstract

Domain adaptation-based models for fault classification under variable working conditions have become a research focus in recent years. Previous domain adaptation approaches generally assume identical label spaces in the source and target domains, however, such an assumption may be no longer legitimate in a more realistic situation that requires adaptation from a larger and more diverse source domain to a smaller target domain with less number of fault classes. To address the above deficiencies, we propose a partial transfer fault diagnosis model based on a weighted subdomain adaptation network (WSAN) in this paper. Our method pays more attention to the local data distribution while aligning the global distribution. An auxiliary classifier is introduced to obtain the class-level weights of the source samples, so the network can avoid negative transfer caused by unique fault classes in the source domain. Furthermore, a weighted local maximum mean discrepancy (WLMMD) is proposed to capture the fine-grained transferable information and obtain sample-level weights. Finally, relevant distributions of domain-specific layer activations across different domains are aligned. Experimental results show that our method could assign appropriate weights to each source sample and realize efficient partial transfer fault diagnosis.

## 1. Introduction

As indispensable parts of rotating machinery, the fault identification and diagnosis of bearings and gears are crucial for the normal operation of the machinery. Since traditional fault diagnosis methods rely on manual processing of vibration signals, it is difficult to explore the depth of fault diagnosis knowledge. With the widely application in industry and academia of deep learning technology, it is possible to mine effective diagnosis knowledge from massive amounts of fault data [1,2,3]. Therefore, such methods have been extensively applied in fault diagnosis of rotating machinery [4,5,6,7].

Li et al. [8] proposed a fault diagnosis framework based on multi-scale permutation entropy (MPE) and multi-channel fusion convolutional neural networks (MCFCNN). Since it considers the structure and spatial information between different sensor measurement points, the fault diagnosis with high accuracy and speed is realized. Valtierra-Rodriguez et al. [9] proposed a methodology based on convolutional neural networks for automatic detection of broken rotor bars by considering different severity levels. This method applies a notch filter to remove the fundamental frequency component of the current signal, and the short-time Fourier transform (STFT) is used to obtain time-frequency plane. Experimental results show that the methods is capable of identifying the healthy condition of the induction motor. However, the distributions of the collected datasets may different due to the change of the operating environments. The diagnostic knowledge in the original training data will no longer be fully applicable to the new testing data when the working condition changes [10,11,12,13,14]. In this case, the fault diagnosis methods under variable working conditions based on transfer learning come into being. Recently, some transfer learning-based methods have been developed to solve cross domain fault diagnosis problem. Mao et al. [15] proposed a deep dual temporal domain adaptation (DTDA) model which could recognize whether an early fault occurs and achieve an earlier detection location and lower false alarm rate. An et al. [16] proposed to apply the maximum mean discrepancy (MMD) based on multiple kernels to intelligent fault diagnosis, and the features of different layers were involved in the domain adaptation process. Wang et al. [17] presented a deep adaptive adversarial network (DAAN) which could narrow the discrepancy to learn domain-invariant features. Chen et al. [18] proposed an unsupervised domain adaptation method which could maximize the mutual information between the target feature space and the entire feature space and minimize the feature-level discrepancy between the two domains. Hasan et al. [19] proposed a multitask-aided transfer learning-based diagnostic framework. This method applies multitask learning-based convolutional network to identify working conditions, and then identifies health status of the rolling element bearings based on transfer learning. In a word, transfer learning techniques provide an efficient solution to cross domain fault diagnosis problems.

Although transfer learning-based methods have made great progress, partial transfer fault diagnosis problem has not been well solved. The partial transfer diagnosis means that the number of fault types in the test data is less than that in the training data. Since the machine is in a healthy working state most of the time, the test data may contain only a few types of fault data. That is, the distribution of two domains is different and the label space of target domain is a subset of that of the source domain [20,21,22]. As many different health types as possible can be involved by training data through a long period of data accumulation, while it is difficult to guarantee the symmetry of health types in testing data and training data. Therefore, this setting is closer to engineering practice compared with the scenario for which the standard domain adaptation is targeted. Since most of the transfer fault diagnosis methods use all source samples for domain adaptation, the unique types of source samples can enable the network to learn false classification knowledge during domain adaptation, which is the major challenge in partial transfer fault diagnosis. Actually, partial transfer problem has been studied in the field of target detection and computer vision. Cao et al. [23] proposed a selective adversarial network (SAN) to facilitate positive transfer by selecting the source samples highly correlated with the target samples. Chen et al. [24] proposed reinforced transfer network (RTNet) which could apply both high-level and pixel-level information to solve partial transfer problem. In addition, importance weighted adversarial nets [25] and example transfer network (ETN) [26] also obtained excellent performance in the image classification task. These works have laid a solid foundation for solving the problem of partial transfer in mechanical fault diagnosis.

Recently, the partial transfer problem has made initial progress in fault diagnosis. Jiao et al. [27] applied weighted cross entropy loss to give smaller weight to the unique source samples, and such weight is determined by the predicted outputs of two classifiers [28]. Li et al. [29] presented a weighted adversarial transfer network (WATN) which used adversarial training to reweight the source domain samples. Yang et al. [30] proposed a deep partial transfer learning network (DPTL-Net) which could learn domain-asymmetry factor to weight the source samples and finally block unnecessary knowledge. The previous partial domain adaptation methods mainly tried to get the weight of the source samples from a global perspective without considering the relationships between two subdomains [31] in source and target domains, which is not conducive to obtaining the fine-grained transferable information in each type of data. To solve the above problem, this paper proposed a weighted subdomain adaptation network (WSAN) to improve the efficiency of partial transfer diagnosis of machinery. All the samples are divided into class-level subdomains, and the subdomain distributions of deep features in multiple layers are aligned. In order to block the samples of outlier source types, an auxiliary classifier is introduced to conduct adversarial training with the feature generator to obtain the class-level weights. To achieve weighted subdomain adaptation, we propose a weighted local maximum mean discrepancy (WLMMD) to measure the Hilbert-Schmidt norm between kernel mean embedding of empirical distributions between relevant subdomains. The main innovations of this work are summarized as follows:

(1) A WSAN framework is presented to solve the partial transfer fault diagnosis problem. Relevant subdomains are built to capture fine-grained transferable information and avoid negative transfer caused by redundant source samples.
(2) The class-level weights are obtained through the adversarial training between the auxiliary classifier and the feature generator. WLMMD is designed to measure the distribution discrepancy between relevant subdomains and obtain fine-grained transferable information. As a result, proper alignment of relevant subdomains in specific activation layers is realized.

The remainder of this work begins with the background of theory in Section 2. In addition, Section 3 provides an introduction to the methodology presented, and Section 4 applies the proposed model to partial transfer fault diagnosis and verifies the advantages of the model by comparing other methods. Finally, some conclusions are drawn in Section 5.

## 2. Theoretical Background

### 2.1. Partial Transfer Fault Diagnosis

**Figure 1:**
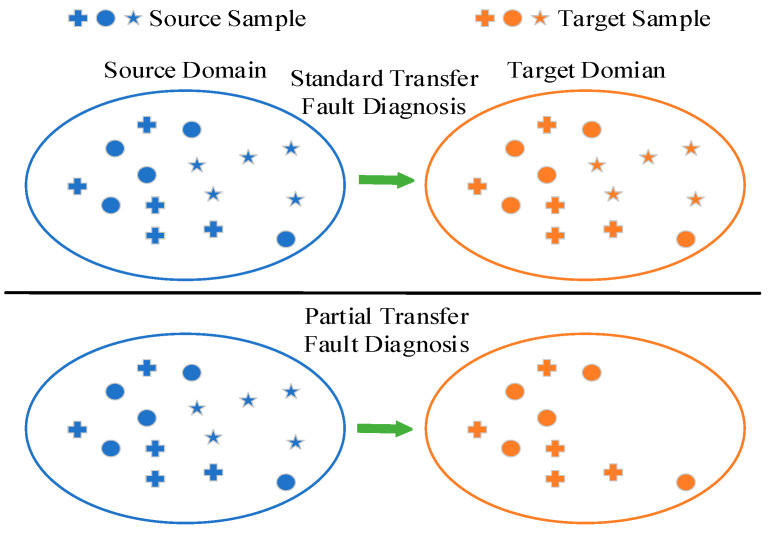
Comparison of standard transfer fault diagnosis and partial transfer fault diagnosis.

For standard domain adaptation-based frameworks, target domain *D_t_* and source domain *D_s_* are collected under different but related working conditions [26]. As shown in the upper part of Figure 1, the job of standard transfer fault diagnosis is to facilitate a knowledge transfer from the labeled source data {*X_s_*, *C_s_*} to the unlabeled target dataset *X_t_*. However, different from the closed transfer fault diagnosis, the source label space *C_s_* and target label space *C_t_* are different in partial transfer diagnosis problem. In the bottom part of Figure 1, there are more source classes than target classes, i.e., $C_{t}\subseteq C_{s}$. In addition, it should be noted that the sample types in the target domain do not deviate from the scope of the source domain, which ensures the authority of the diagnostic knowledge in source domain. The purpose of partial transfer fault diagnosis is to find the categories associated with the source domain and classify them accurately.

### 2.2. Subdomain Adaptation

**Figure 2:**
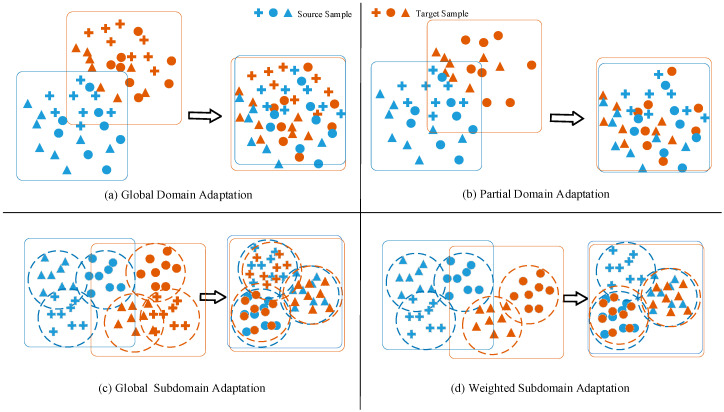
Comparison of standard domain adaptation (**a**,**b**) and subdomain adaptation (**c**,**d**). The box represents the data distribution range and the arrows represent the domain or subdomain adaptation process. The dotted circles represent the divided subdomains.

The source and target domains may consist of some subdomains that can be defined according to different criteria, such as class or category. For partial transfer fault diagnosis, the number of sample types in the source domain must be no less than that in the target domain, so is practicable to delimit the subdomains based on the number of types in the source domain, although this may not be appropriate for the target domain, but it ensures alignment of local data distribution discrepancy. As can be seen from Figure 2a,b, it is difficult to match two data distributions directly in the process of global or partial domain adaptation. In Figure 2c,d, subdomain adaptation is of superior feature representation ability because the fine-grained transferable information within the subdomains is utilized [31]. However, the problem with this is that the data in the target domain is unlabeled, which prevents target domain from being partitioned. Fortunately, we take the prediction probability output of the model for the target samples as pseudo-labels to divide them into some subdomains. In this way, subdomain adaptation enables the model to focus more on local data distribution differences.

### 2.3. Weighted Local Maximum Mean Discrepancy

In the field of transfer learning, MMD [32] is a common nonparametric metric that measures the discrepancy between two distributions. It takes the mean embeddings of two distributions in a Reproducing Kernel Hilbert Space (RKHS) as a distance calculation to avoid the density estimation. MMD can be defined as:

(1) $$d_{ℋ}(D_{s},D_{t})≜{\left\|E_{p}\left[\phi \left(x^{s}\right)\right]-E_{q}\left[\phi \left(x^{t}\right)\right]\right\|}_{ℋ}^{2},$$

where ϕ(·) is the feature mapping function that maps the original data to RKHS ℋ. Therefore, an estimate of the MMD compares the square distance between the empirical kernel mean embeddings as:

(2) $$\begin{array}{cc} {\hat{d}}_{ℋ}(D_{s},D_{t}) & ={\left\|\frac{1}{n_{s}}\sum_{x_{i}\in D_{s}}\phi \left(x_{i}\right)-\frac{1}{n_{t}}\sum_{x_{j}\in D_{t}}\phi \left(x_{j}\right)\right\|}_{ℋ}^{2}\\ & =\frac{1}{n_{s}^{2}}\sum_{i=1}^{n_{s}}\sum_{j=1}^{n_{s}}k\left(x_{i}^{s},x_{j}^{s}\right)+\frac{1}{n_{t}^{2}}\sum_{i=1}^{n_{t}}\sum_{j=1}^{n_{t}}k\left(x_{i}^{t},x_{j}^{t}\right)\\ & -\frac{2}{n_{s}n_{t}}\sum_{i=1}^{n_{s}}\sum_{j=1}^{n_{t}}k\left(x_{i}^{s},x_{j}^{t}\right) \end{array}$$

where ${\hat{d}}_{H}\left(p,q\right)$ is an unbiased estimator of $d_{H}\left(p,q\right)$. *n_s_* and *n_t_* are the number of source samples and target samples, respectively.

Most previous domain adaptation methods apply MMD to narrow the distribution discrepancy without considering the internal distribution of the data. However, such methods may result in poor alignment because the relationship between related subdomains is ignored. Furthermore, these methods also fail to selectively involve source samples in the adaptation process due to the asymmetry of data types across the two domains. Considering the above problems, we propose the WLMMD to achieve weighted subdomain adaptation:

(3) $$d_{ℋ}(D_{s},D_{t})≜E_{c}∥E_{p^{\left(c\right)}}\left[\phi \left(x^{s}\right)\right]-E_{q^{\left(c\right)}}\left[\phi \left(x^{t}\right)\right]{∥}_{ℋ}^{2},$$

where ***x**^s^* and ***x**^t^* are the instances in *D_s_* and *D_t_*, and *p^(c)^*, and *q^(c)^* are the distributions of $D_{s}^{\left(c\right)}$ and $D_{t}^{\left(c\right)}$, respectively. So we can calculate an unbiased estimator of WLMMD as:

(4) $${\hat{d}}_{ℋ}(D_{s},D_{t})=\frac{1}{C}\overset{C}{\underset{k=1}{\sum }}∥\underset{x_{i}^{s}\in D_{s}}{\sum }w_{i}^{sk}\phi \left(x_{i}^{s}\right)-\underset{x_{j}^{t}\in D_{t}}{\sum }w_{j}^{tk}\phi \left(x_{j}^{t}\right){∥}_{ℋ}^{2},$$

where $w_{i}^{sk}$ and $w_{j}^{tk}$ denote the weights of $x_{i}^{s}$ and $x_{i}^{t}$ belonging to class *k*, respectively. Obviously, $\sum_{i=1}^{n_{s}}w_{i}^{sk}=\sum_{i=1}^{n_{t}}w_{i}^{tk}$ = 1, and $w_{i}^{k}$ for the sample *x_i_* can be computed as:

(5) $$w_{i}^{k}=\frac{y_{ik}}{\sum_{\left(x_{j},y_{j}\right)\in D}y_{jk}},$$

where *y_ic_* is the *k*-th entry of vector ***y**_i_*. Since the source samples are labeled with a one-hot vector, we can directly calculate the weight $w_{i}^{sk}$ by the labels. Although the samples of the target domain are unlabeled, it is feasible to use pseudo labels to partition related subdomains. Note that the predicted output ${\hat{y}}_{i}^{t}$ given by the classifier can be used as pseudo target labels which measures the probability that the target sample belongs to the corresponding category. ${\hat{y}}_{i}^{t}$ can be regarded as the probability of assigning $x_{i}^{t}$ to each of the C classes, and the weight $w_{i}^{tk}$ of target samples could be acquired. Thus, we can approximate Equation (5) as:

(6) $$\begin{array}{cc} {\hat{d}}_{l}(D_{s},D_{t})=\frac{1}{C}\sum_{k=1}^{C} & [\sum_{i=1}^{n_{s}}\sum_{j=1}^{n_{s}}w_{i}^{sk}w_{j}^{sk}k\left(z_{i}^{sl},z_{j}^{sl}\right)\\ & +\sum_{i=1}^{n_{t}}\sum_{j=1}^{n_{t}}w_{i}^{tk}w_{j}^{tk}k\left(z_{i}^{tl},z_{j}^{tl}\right)\;\\ & -2\sum_{i=1}^{n_{s}}\sum_{j=1}^{n_{t}}w_{i}^{sk}w_{j}^{tk}k\left(z_{i}^{sl},z_{j}^{tl}\right)] \end{array}$$

where ***z****^l^* is the *l*th layer activation of *L* layers. By using Equation (6), the distribution discrepancy between the two subdomains at a particular activation layer can be calculated.

## 3. Proposed Method

### 3.1. Weighted Subdomain Adaptation Network

**Figure 3:**
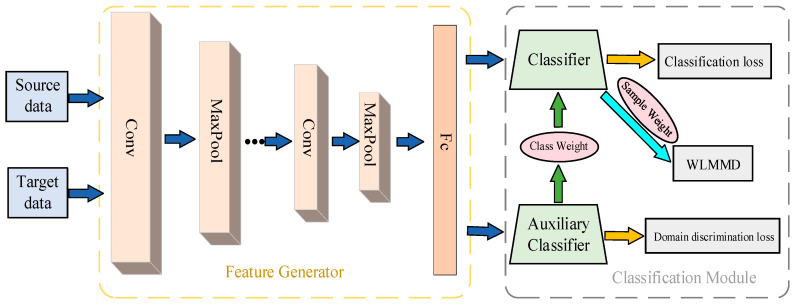
The structural composition of the proposed model.

In order to achieve efficient partial transfer fault diagnosis, we design a novel weighted subdomain adaptation network (WSAN). The details of the proposed model are clearly presented in Figure 3. The feature generator *G* is a deep structure based on one dimensional convolutional neural network (1D-CNN) that is expected to extract domain invariant deep features. The auxiliary classifier *C_A_* is set to obtain the class-level weights of the source samples, which is achieved by adversarial training. After acquiring class-level weights, weighted subdomain adaptation can be carried out in activation layers of the classifier *C* based on WLMMD. The objective function can be written as:

(7) $$\begin{array}{cc} F_{0}\left(\theta_{G},\theta_{C}\right) & =\frac{1}{n_{s}}\sum_{i}^{n_{s}}L_{c}\left(G\left(x_{si}^{}\right),y_{si}^{}\right)\\ & -\frac{\lambda_{0}}{n_{s}+n_{t}}\sum_{x_{i}\in \left(D_{s}\cup D_{t}\right)}L_{d}\left(D\left(G\left(x_{i}\right)\right),d_{i}\right)\\ & +\gamma_{0}{\hat{d}}_{l}(D_{s},D_{t}) \end{array}$$

where *λ*_0_ and *γ*_0_ are the penalty coefficients, *y_si_* and *d_i_* are the source sample label and domain label, and *L* denotes the condition prediction loss.

### 3.2. Adversarial Training-Based Class-Level Weights Obtaining

Due to the asymmetry of the fault classes in the two domains, samples of redundant types in the source domain may cause a negative transfer. Therefore, these redundant subdomains must be selected to block the classification knowledge that is unfavorable to the recognition of target samples. Inspired by generative adversarial networks (GAN), we set up an auxiliary classifier *C_A_* to play the mini-max game with the feature generator. Specifically, given input ***x****_s_* or ***x****_t_* with the label 1 or 0, after multiple layers of extraction, the feature generator *G* narrows the domain shift to make classifier cannot distinguish the true source of the input sample. The auxiliary classifier is trained to give the correct label. The objective of the adversarial training can be defined as:

(8) $$\begin{array}{cc} \underset{G}{\min}\underset{C_{A}}{\max}ℒ\left(C_{A},G\right) & =\frac{1}{n_{s}}\sum_{i=1}^{n_{s}}\log\left(C_{A}\left(G(x_{si}^{})\right)\right)\\ & +\frac{1}{n_{t}}\sum_{j=1}^{n_{t}}\log\left(1-C_{A}\left(G(x_{tj}^{})\right)\right) \end{array}$$

The distribution differences of the deep features of shared fault types will be narrowed in the training process, so the auxiliary classifier will be unable to distinguish samples of these types and give an output close to 0, while the output of the unique source samples will be close to 1. The aim of adversarial training is to learn the relative importance of source samples, suggesting that the outlier samples should be assigned a relatively small weight. Therefore, the weight function is inversely related to *C_A_*(*G*(***x***)) and the importance weights function can be defined as:

(9) $$w_{c}(x)=1-C_{A}\left(G(x)\right),$$

After obtaining the class weights of the source samples, the overall objective can be rewritten as:

(10) $$\begin{array}{cc} F\left(\theta_{G},\theta_{C}\right) & =\frac{1}{n_{s}}\sum_{i}^{n_{s}}\sum_{x_{si}\in D_{s}^{j}}w_{c}{}_{j}L_{c}\left(G\left(x_{si}^{}\right),y_{si}^{}\right)\\ & +\frac{\lambda_{1}}{n_{s}}\sum_{i=1}^{n_{s}}\sum_{x_{si}\in D_{s}^{j}}w_{c}{}_{j}\log\left(C_{A}\left(G(x_{si}^{})\right)\right)\\ & +\frac{\lambda_{2}}{n_{t}}\sum_{j=1}^{n_{t}}\log\left(1-C_{A}\left(G(x_{tj}^{})\right)\right)\\ & +\gamma {\hat{d}}_{l}(D_{s},D_{t}) \end{array}$$

(11) $$\begin{array}{cc} F\left(\theta_{C_{A}}\right)= & -\frac{1}{n_{s}}\sum_{i=1}^{n_{s}}\sum_{x_{si}\in D_{s}^{j}}w_{c}{}_{j}\log\left(C_{A}\left(G(x_{si}^{})\right)\right)\\ & -\frac{\lambda }{n_{t}}\sum_{j=1}^{n_{t}}\log\left(1-C_{A}\left(G(x_{tj}^{})\right)\right) \end{array}$$

where *w_cj_* and $D_{s}^{j}$ denote the weights and samples for the *j*-th source class, *y_s_* is the source sample labels and *γ* is a penalty coefficient.

## 4. Experiments

### 4.1. Dataset Introduction

**Figure 4:**
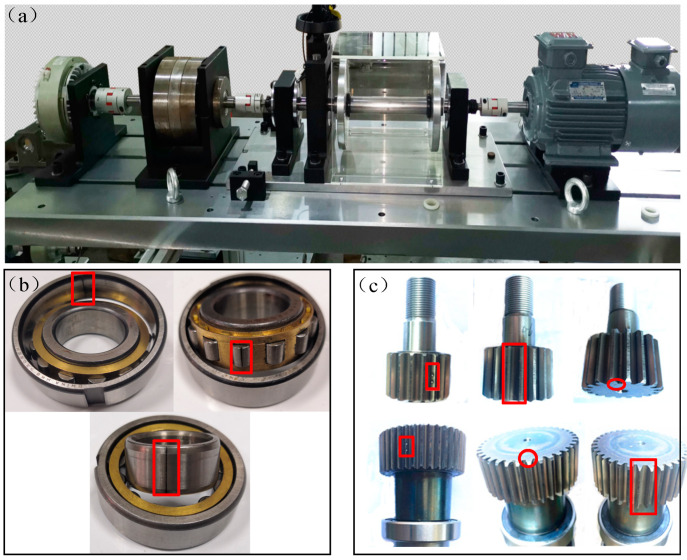
Representation for rotating machinery fault diagnosis test bed in our laboratory (**a**) and different types of bearing fault (**b**) and gear fault (**c**). Red box indicates the location of damage.

The proposed framework is verified with the datasets collected in our laboratory to validate the performance in partial transfer fault diagnosis. Figure 4a indicates the experimental equipment used in our laboratory. The platform consists of a motor, two balancing rotors, two bearing seats, a planetary gearbox, and a magnetic brake for controlling load. Vibration sensors are installed on fixed holders at both ends of the gearbox, and the sampling frequency is 25.6 kHz.

(1) Bearing fault dataset

Five health conditions are involved in the bearing fault dataset, namely, normal, inner ring fault, outer ring fault, rolling element fault, and combined fault of the rolling element and outer ring. The fault parts are shown in Figure 4b. There are two damage sizes for each type of fault, specifically, 0.2 and 0.4 mm. Thus, the bearing fault dataset contains samples of nine health types, namely, NC, IF1, IF2, OF1, OF2, RF1, RF2, RO1, and RO2. Four datasets are obtained under different loads, specifically, L1 (80 N), L2 (60 N), L3 (40 N), and L4 (20 N). And the engine speed is 2000 r/min.

(2) Gear fault dataset

As shown in Figure 4c, the gear fault dataset contains samples of seven health types, namely, normal condition (NC), sun gear fracture (SF), sun gear pitting (SP), sun gear wear (SW), planet gear fracture (PF), planet gear pitting (PP), and planet gear wear (PW). We collected three datasets at different rotational speeds (without load), specifically, S1 (2200 r/min), S2 (2000 r/min), and S3 (1800 r/min).

The number of each type of samples is 500. Thus, the number of samples in bearing and gear datasets are 4500 and 3500, respectively. In order to give full play to the feature extraction and weight learning ability of the proposed method, 40% of the samples were used for training and the remaining for testing.

### 4.2. Compared Methods

To show the superior performance of the proposed model, four comparative methods are adopted as follows:

(1) Supervised training without classification knowledge transfer is adopted as a basic comparative method (Basic), and it obtained classification knowledge only from the source domain samples.
(2) Domain adaptation framework based on multiple kernel variant of maximum mean discrepancy (MKMMD) [16]: Efficient kernel method is adopted in different layers of the network, and excellent performance was achieved on the global domain adaptation task.
(3) Deep subdomain adaptation network (DSAN) [31]: As a typical global domain adaptation approach, it does not include class-level weight acquisition, that is, the auxiliary classifier is not adopted in the network. In this method, local maximum mean difference (LMMD) is used for effective subdomain adaptation.
(4) Example transfer network (ETN) [26]: It is an adversarial discriminative domain adaptation method, and the adversarial training is adopted to obtain the weights of the source samples. Similar to the proposed method, an auxiliary domain discriminator and an auxiliary classifier are adopted to obtain the sample weights in the source domain.

### 4.3. Implementation Details

As detailed in Table 1, we randomly discarded a number of fault types to design different partial transfer diagnosis tasks on the basis of the two fault diagnosis datasets. For the dataset, each sample consists of 2400 data points, then fast Fourier transformation (FFT) is applied to transform the time-domain signal to frequency-domain signal that contains 1200 Fourier coefficients. The structure of the framework are illustrated in Table 2. The learning rate is set as 0.0001, and the maximum training epoch is 1000. In order to avoid the effects of random cause, we conducted 10 experiments on each task. The running steps of the proposed model are shown in Algorithm 1. In the test process, the spectral data of the target domain can be directly input into the model for classification. The code programming of the model is implemented on the Pytorch platform.
**Algorithm 1:** Weighted Subdomain Adaptation Network (WSAN)        **Model:** Feature generator *G*; Auxiliary classifier *C_A_*; Classifier *C*.        **Input:** Labeled source data {*X_s_*, *C_s_*} and unlabeled target data *X_t_*.        For ***i*** in **epochs**:            **Step 1**: The feature generator *G* outputs the high-dimensional features of the two domains and inputs them into the feature generator *G* and classifier *C*.            **Step 2**: Auxiliary classifier *C_A_* obtains the class-level weights. The classifier gives prediction probability output on the target samples and obtains sample-level weights to guide WLMMD to perform subdomain adaptation.            **Step 3**: Train the feature generator *G* and classifier *C* to obtain the optimal parameters ${\hat{\theta }}_{G}$ and ${\hat{\theta }}_{C}$ by minimizing *F*($\theta_{G},\theta_{C}$);            **Step 4:** Train the auxiliary classifier *C_A_* to obtain the optimal parameters ${\hat{\theta }}_{C_{A}}$ by minimizing *F*($\theta_{C_{A}})$;

### 4.4. Experimental Results

**Figure 5:**
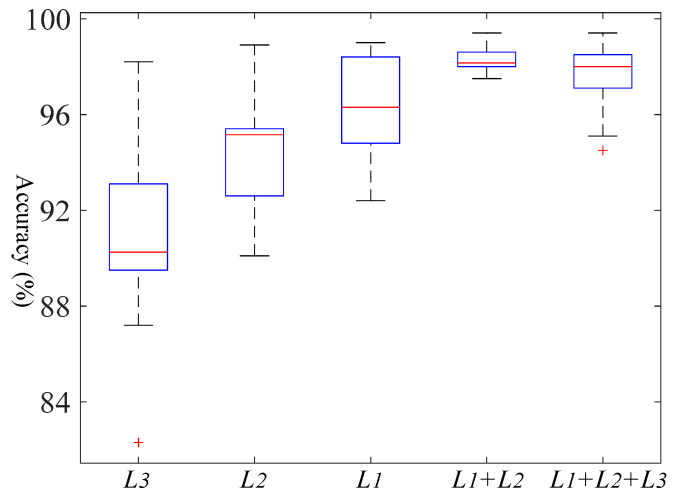
Boxplot for the performance of different layer combinations under the same task.

As mentioned in Section 2, the deep features in different activation layers of the model are involved in subdomain adaptation. In order to obtain the best performance for subdomain adaptation, the deep features with dimensions 128, 256 and 512 in fully connected layers of the classifier and feature generator (named L1, L2 and L3, respectively) are extracted and combined for comparison. B7 task was selected to verify the combination of different layers, and the experiment was conducted for 15 times. As shown in Figure 5, it is clear that L1 achieves the best performance during the single layer, while L3 performs the worst. It means that the model needs to carry out deep operation to extract more separable domain invariant features. In the multi-layer combination, L1 + L2 performed better than single layer while L1 + L2 + L3 has a lower performance than L1 + L2. This indicates that some non-invariant features may exist in the shallow layers and using subdomain adaptation to align these features will degrade the performance of the model. Therefore, we apply the combination of L1 + L2 for the designed tasks.

The average accuracy of the proposed method and the comparison method in all tasks are detailly shown in Table 3. In general, our method obtains the highest average accuracy and the lowest standard deviation. This indicates that WSAN has excellent and stable performance in both global and partial domain adaptation tasks. Since the basic approach does not include any domain adaptation operations, it obtains the worst performance on all tasks. MKMMD achieves the highest accuracy on the non-partial transfer fault diagnosis task B1, but performed poorly on the partial transfer tasks. This indicates that the domain adaptation methods based on MMD has superior performance in the fault diagnosis task under variable conditions, but it is not feasible to directly apply it to partial transfer scenarios. DSAN performed better than MKMMD in most tasks, and its average accuracy is 4.2% higher than that of MKMMD. But it still lags behind the other two partial transfer methods because it does not carry out any weight learning operation. WSAN achieved an average accuracy of 97.7%, which was 4.7% higher than ETN, 13.2% higher than DSAN, and 17.4 higher than MKMMD.

It can be noted that ETN and WSAN, as two domain adaptation methods with weighted learning, perform significantly better than other methods in partial transfer diagnosis tasks. In addition, it can be found that the proposed method gets more ahead of ETN with the increasing degree of domain class asymmetry. For task B2, the accuracy of WSAN is 1.4% higher than that of ETN, while WSAN is 5.6% higher than that of ETN on task B8. The same phenomenon can be observed for tasks G1 and G6.

**Figure 6:**
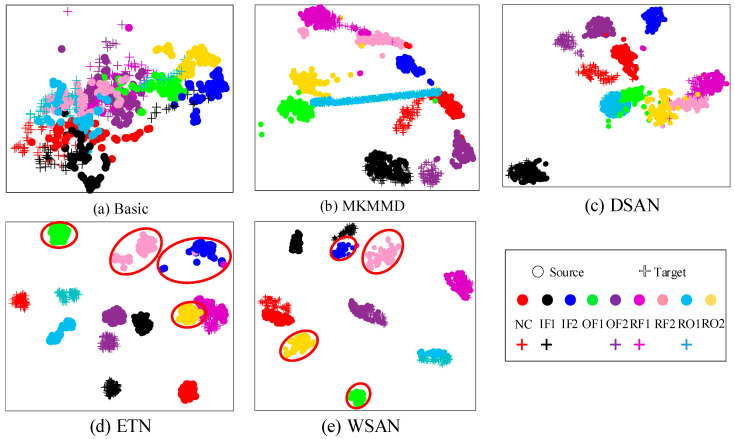
*t*-SNE visualization results of (**a**) Basic, (**b**) MKMMD, (**c**) DSAN, (**d**) ETN, and (**e**) WSAN in task B3. The samples circled in red are outlier types.

To demonstrate the feature classification effect of our method intuitively, the high-dimensional features extracted of the model are processed with the well-known *t*-SNE [33] technology for dimension reduction. The dimension reduction results of B3 are shown in Figure 6. In Figure 6a, we can see that the feature separability and clustering effect obtained by the basic method are inefficient. Features become separable but shared types and outliers are still cannot be distinguished in Figure 6b,c when domain adaptation is adopted. Although MKMMD and DSAN perform efficient global domain adaptation, the existence of outlier types would enable the model to extract classification knowledge that is not applicable in the target domain. This also indicates that the global adaptation methods only pays attention to the alignment of the two domains, but does not consider the relationship between the subdomains within the domain. In Figure 6d, it can be seen that ETN basically separates outlier samples but the alignment of shared type features is not accurate enough, which indicates that the classifier cannot carry out effective sample-level alignment after obtaining class-level weights and it may leads to inaccurate classification. There are some confusions between the source samples of RO2 and RF1 types. In this case, ETN may treat the RF1 samples as outliers and filter out some useful classification knowledge. For the proposed method, precise alignment of the related subdomains is performed while blocking the outlier types in Figure 6e. After obtaining accurate class-level weights, WSAN can use the proposed WLMMD to perform effective subdomain alignment which involves the sample-level weights learning.

**Figure 7:**
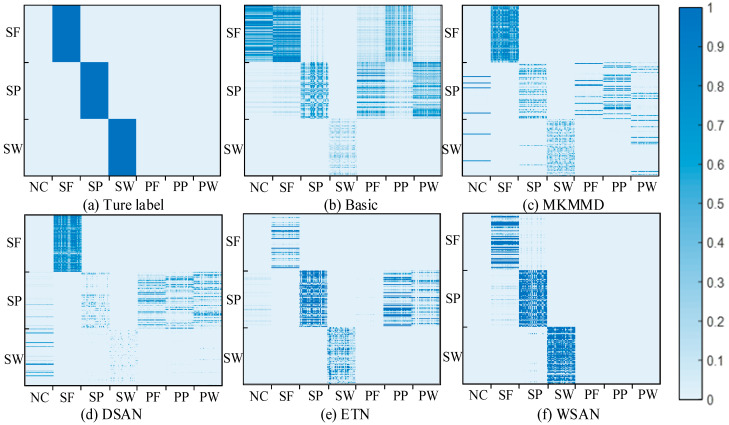
Similarity matrix of learned features by (**a**) ture label, (**b**–**e**) comparison methods, and (**f**) the proposed method. The abscissa and ordinate represent the source sample sequence and target sample sequence, respectively. The depth of the color indicates the similarity between the corresponding samples.

In order to further explore how the weights learned affect the alignment of deep features, the similarity matrix of source and target features in deep layer is drawn on task G4. According to [30], the similarity matrix can be calculated by *G*(***x****_i_*,***x****_j_*) = exp(−‖***x****_i_* − ***x****_j_*‖^2^/200) wherein ***x****_i_* ∈ *D^s^* and ***x****_j_* ∈ *D^t^*. Figure 7a shows the actual correspondence between the source and target labels. In Figure 7b, only the samples of SW type can be identified to a certain extent, while the features extracted from the other two target types of samples are highly similar to various source types, which is extremely unfavorable for classification. Obviously, the deep features extracted by the basic method are chaotic due to the lack of domain adaptation operation. In Figure 7c,d, the corresponding samples of SP and SW types have low similarity degree, and some of the samples have great similarity with other types. Consequently, global domain adaptation methods may extract fuzzy deep features when dealing with partial transfer problem. Figure 7e shows that ETN can assign large weight to shared types, but there are still some outlier samples with large weights, resulting in a higher similarity between target features of SP and source features of PP and PW. By comparison, Figure 7f indicates that WSAN obtains more accurate weights, which is reflected in the large similarity between the extracted features of the target domain and corresponding features of source domain, and only a few samples are weakly similar to other source types. In general, the proposed method can make the shared samples fully participate in the subdomain adaptation and block outliers. Thus, the extracted domain invariant features own high similarity among the corresponding shared types.

## 5. Conclusions

A weighted subdomain adaptation network (WSAN) is presented to solve partial transfer fault diagnosis problem of machinery. Different from the previous global domain adaptation approaches, we divide all samples into different subdomains according to sample types of the source domain, and design WLMMD to perform accurate subdomain alignment. In addition, in order to obtain class-level weights, an additional auxiliary classifier is set up to conduct adversarial training with the feature generator. Under the guidance of class-level weights, the prediction probability output of the target domain by the classifier is used as the sample-level weights, so that the model could capture fine-grained transferable information within the relevant subdomains. The optimal layer combination was found by exploring the performance of the deep features in different activation layers participating the subdomain adaptation. The best diagnostic performance can be obtained under the combination of fully connected layers (L1 + L2) with dimensions 128 and 256. Experimental results on the bearing and gear datasets collected in our laboratory indicates that the average accuracy of the proposed method on the designed fault diagnosis task is 97.7%, which is higher than that of several comparison methods. This means WSAN could solve the partial transfer fault diagnosis problem more efficiently compared several popular methods. *t*-SNE dimension reduction and correlation matrix show that WSAN can learn accurate weights and carry out accurate weighted subdomain adaptation.

Although the proposed weighted subdomain adaptation approach achieves superior performance on the partial transfer fault diagnosis tasks, the laboratory works on the premise that the target data is available during training. It is difficult to guarantee the performance of such a model under unknown working conditions. Such approaches may fail when we need real-time diagnosis. However, this problem may be solved with the help of domain generalization technology [34], and we will explore this issue in depth in our future work.

## Figures and Tables

**Table 1:** Descriptions of the diagnosis tasks.

Dataset	Task	Transfer	Target	Source
Bearing	B1	L1→L2	All types	All types
	B2	L1→L3	NC, IF1, IF2, OF1, OF2, RF1, RF2, RO2	
	B3	L1→L4	NC, IF1, IF2, OF2, RF1, RO2	
	B4	L2→L3	NC, IF1, OF2, RF1, RO1	
	B5	L2→L1	NC, OF1, OF2, RO1	
	B6	L3→L4	IF1, OF1, RF1	
	B7	L3→L1	IF1, IF2	
	B8	L4→L3	RO2	
Gear	G1	S1→S2	NC, SF, SP, SW, PF, PP	All types
	G2	S1→S3	NC, SF, SP, SW, PF	
	G3	S2→S3	SF, SP, SW, PF	
	G4	S2→S1	SF, SP, SW	
	G5	S3→S1	NC, SP	
	G6	S3→S2	PW	

**Table 2:** Structural composition of the proposed model.

Module	Layer	Size	Channels × Kernel Size	Stride	Activation
Feature Generator	Input data	1200	/	/	/
	Conv	8 × 300	8 × 3	4	ReLu
	MaxPool	8 × 150	8 × 2	2	/
	Conv	16 × 150	16 × 3	1	ReLu
	MaxPool	16 × 75	16 × 2	2	/
	Conv	32 × 75	32 × 3	1	ReLu
	MaxPool	32 × 37	32 × 2	2	/
	Conv	32 × 37	32 × 2	1	ReLu
	MaxPool	32 × 17	32 × 2	2	/
	Fc	512	/	/	/
Classifier	Fc	256	/	/	/
	Fc	128	/	/	/
	Fc	*C*	/	/	Softmax
Auxiliary Classifier	Fc	256	/	/	/
	Fc	128	/	/	/
	Fc	1	/	/	Sigmoid

**Table 3:** Experimental results of the average testing accuracies in all tasks (%).

Task	Basic	MKMMD	DSAN	ETN	WSAN (Ours)
B1	73.1 (±2.1)	99.5 (±0.1)	98.3 (±0.3)	98.8 (±0.2)	**99.4 (±0.1)**
B2	65.5 (±4.5)	90.1 (±0.3)	92.6 (±0.8)	97.1 (±0.3)	**98.5 (±0.1)**
B3	63.7 (±3.4)	87.2 (±0.8)	90.3 (±1.1)	95.5 (±0.5)	**98.6 (±0.2)**
B4	59.1 (±5.8)	80.4 (±2.1)	90.1 (±1.5)	92.5 (±0.6)	**98.5 (±0.2)**
B5	59.7 (±4.6)	82.3 (±2.0)	85.4 (±2.6)	93.0 (±0.7)	**97.9 (±0.2)**
B6	64.0 (±5.8)	79.6 (±3.9)	80.1 (±3.9)	91.4 (±1.0)	**98.0 (±0.1)**
B7	45.2 (±8.1)	68.2 (±7.3)	75.2 (±5.4)	88.5 (±1.1)	**97.8 (±0.3)**
B8	42.4 (±6.5)	63.1 (±8.0)	78.9 (±7.2)	92.4 (±0.6)	**98.0 (±0.2)**
G1	60.2 (±4.4)	88.1 (±0.3)	90.6 (±0.5)	98.4 (±0.6)	**99.2 (±0.1)**
G2	55.1 (±5.0)	85.2 (±0.8)	90.0 (±1.1)	92.5 (±0.9)	**97.1 (±0.1)**
G3	50.7 (±5.3)	80.4 (±2.1)	84.1 (±1.5)	94.3 (±1.7)	**98.2 (±0.2)**
G4	52.0 (±3.8)	82.3 (±2.0)	85.4 (±2.6)	92.4 (±2.0)	**95.6 (±0.3)**
G5	41.2 (±4.0)	69.6 (±3.9)	71.2 (±3.5)	88.5 (±1.8)	**95.1 (±0.3)**
G6	35.4 (±5.6)	68.2 (±5.3)	70.2 (±4.0)	88.1 (±0.9)	**95.5 (±0.6)**
Average	55.2	80.3	84.5	93.0	**97.7**

## Data Availability

The data presented in this study are available on request from the corresponding author.
